# Genetic characterization of *Toxoplasma gondii* from cats in Yunnan Province, Southwestern China

**DOI:** 10.1186/1756-3305-7-178

**Published:** 2014-04-11

**Authors:** Yi-Ming Tian, Si-Yang Huang, Qiang Miao, Hai-Hai Jiang, Jian-Fa Yang, Chunlei Su, Xing-Quan Zhu, Feng-Cai Zou

**Affiliations:** 1 College of Animal Science and Technology, Yunnan Agricultural University, 650201 Kunming, Yunnan Province, PR China; 2 State Key Laboratory of Veterinary Etiological Biology, Key Laboratory of Veterinary Parasitology of Gansu Province, Lanzhou Veterinary Research Institute, Chinese Academy of Agricultural Sciences, 730046 Lanzhou, Gansu Province, PR China; 3 Department of Microbiology, The University of Tennessee, 37996 Knoxville, TN, USA; 4 School of Life Sciences, Yunnan University, 650091 Kunming, Yunnan Province, PR China

**Keywords:** *Toxoplasma gondii*, Cats, Genetic characterization, Multilocus PCR-RFLP, Yunnan

## Abstract

**Background:**

Cats are the definitive hosts of *Toxoplasma gondii*. The distribution of genetic diversity of *T. gondii* in cats is of importance to understand the transmission of this parasite. The objective of this study was to genetically characterize *T. gondii* isolates from cats in Yunnan province, southwestern China.

**Methods:**

Genomic DNA was extracted from 5–10 g cat tissue samples (brain, tongue, heart, and liver). Using multilocous polymerase chain reaction-restriction fragment length polymorphism (PCR-RFLP) technology, we determined genetic diversity of *T. gondii* isolates from cats in Yunnan province.

**Result:**

In total, 175 stray cats were tested for *T. gondii* DNA, respectively, 44 (25.14%) of which were found to be positive for the *T. gondii* B1 gene by PCR amplification. The positive DNA samples were typed at 11 genetic markers, including 10 nuclear markers, namely, SAG1, 5*'*-3*'*SAG2, alternative SAG2, SAG3, GRA6, L358, PK1, BTUB, c22-8, c29-2 and an apicoplast locus Apico. Of these, 16 isolates from cats were genotyped with data for more than 9 loci, revealed 5 genotypes in total, of which 11 of 16 samples were identified as ToxoDB#9, two samples may belong to genotye #225, one was Type II, one was ToxoDB#3, and one was ToxoDB#20 (http://toxodb.org/toxo/).

**Conclusions:**

The results of the present study indicated a wide distribution of *T. gondii* infection in cats in Yunnan province, which may pose significant public health concerns. To our knowledge, the present study is the first report of *T. gondii* prevalence and genotypes in cats in southwestern China, and the first report of Type II *T. gondii* from cats in China.

## Background

*Toxoplasma gondii* is an obligate intracellular parasite that has a remarkable ability to infect almost all warm-blooded animals, including humans [1]. It is transmitted to humans through consumption of undercooked meat containing *T. gondii* tissue cysts, or by food or water contaminated with oocysts shed in the feces of infected cats. *T. gondii* establishes a lifelong chronic infection in the host. Though the infection rarely causes clinical symptoms in healthy adults, it can be fatal in immunocompromised individuals, such as AIDS patients or patients undergoing immunosuppressive therapy [2]. Cats are the only known animals that serve as the definitive hosts where sexual multiplication of the parasite occurs, resulting in excreting oocysts into the environment which may be a potential source of infection for all types of warm-blooded animals [1].

*T. gondii* strains isolated from Europe and North America belong to three distinct clonal lineages, Types I, II, and III, which differ in many phenotypes, including pathogenicity, and a fourth clonal lineage (type 12) in North America was identified in wildlife recently [3]. However, *T. gondii* strains from South America are genetically more diverse [4-6].

China is a large country, but limited information concerning genetic characterization of *T. gondii* isolates from cats is available [7-11]. The climate in China differs from region to region because of the country’s highly complex topography, thus, the genetic diversity of *T. gondii* isolates could be different. Yunnan is a province located in the far southwest of the country, it has complex topography and diverse climate. Seroprevalence rates of *T. gondii* infection in this province are 35%, 24%, 22%, 17% and 13% for HIV positive patients, HIV negative control, pet dogs, pigs and peafowls, respectively, indicating wild distribution of *T. gondii* infection in this region [12-15]. However, there is no epidemiology or genotype information on *T. gondii* in cats here. Genetic analysis of *T. gondii* infection in cats is of importance to understand the epidemiology, transmission patterns and mechanisms of the disease. Thus, the objectives of this study were to determine the prevalence and genetically characterize *T. gondii* in cats in Yunnan province, southwestern China.

## Methods

### Ethics statement

The present study was approved by the Animal Ethics Committee of Lanzhou Veterinary Research Institute, Chinese Academy of Agricultural Sciences (Approval No: LVRIAEC2011-007). All cats were handled in strict accordance with good animal practice according to the Animal Ethics Procedures and Guidelines of the People’s Republic of China.

### Sample collection

A total of 175 stray cats were collected randomly from Yunnan province during June 2011 to June 2012, these cats came from different cities of Yunnan, including 43 from Jinping, 23 from Nujiang, 4 from Kunming, 54 from Eryuan, 13 from Banna, and 38 from Yimen. Most of the cats were collected dead, thus, the information about breed and age of these cats were not available. Tissue samples (brain, tongue, heart, and liver) were collected from the cats for *T. gondii* detection. All tissue samples were stored at −20°C prior to use.

### Genomic DNA extraction

Genomic DNA was extracted from cat tissue samples using TIANamp Genomic DNA kit (TianGen™, Beijing, China) according to manufacturer’s recommendations. In brief, 30 mg of each tissue were treated with sodium dodecyl sulphate/proteinase K at 56°C for overnight digestion in thermostat water bath. DNA samples were prepared after purification by silica gel column chromatography and eluted into 50 μL elution buffer.

### Genetic characterization of *T. gondii* isolates

The DNA samples from cat tissues were first screened for *T. gondii* infection using semi-nested PCR of the B1 gene [16] and then the positive samples were genotyped using Multi-locus PCR-RFLP (Mn-PCR-RFLP) method [17]. In brief, the target DNA sequences were amplified by multiplex PCR using external primers for all 11 markers. The reaction volume consisted of 25 μl containing 100 ng genomic DNA with positive control samples. Nine *T. gondii* strains were included as the positive controls (Table 1). The PCR reaction composed of 1× PCR buffer, 0.2 mM of each primer, 200 μM dNTPs, 2 mM MgCl_2_, 0.2 U of HotStart Taq DNA polymerase (TAKARA, Japan). The PCR amplification was performed using thermal cycler (PTC 200, Bio-RAD). All samples were incubated at 95°C for 5 min to activate the DNA polymerase, then 30 cycles of PCR at 95°C for 30 s, 55°C for 60 s and 72°C for 90 s. Then 1 μL of the products served as template DNA for nested PCR with internal primers for each marker, respectively. A similar program was used for the nested PCR. The nested PCR was carried out with an annealing temperature at 60°C for 60 s for all the markers except Apico, which was amplified at 55°C. The nested PCR products were digested with restriction enzymes for 1 h, and the temperature for each enzyme followed the instruction for each enzyme. The restriction fragments were resolved in 2.5%-3% agarose gel to display DNA fragment length polymorphism using a gel document system (UVP GelDoc-It™ Imaging System, Cambridge, U.K.).

### Statistical analyses

Results were analyzed with SPSS for Windows (Release18.0 standard version, SPSS Inc., Chicago, Illinois). Generalized Lineal Model (GLM) test was used to analyze the prevalence of *T. gondii* in different regions and tissues. The differences were considered to be statistically significant when the P-value was less than 0.05.

## Results and discussion

Forty-four (25.14%) out of 175 cats were *T. gondii* B1 gene positive detected by PCR. The positive samples were distributed in all six administrative cities with the prevalence ranging from 14.81% (Eryuan) to 39.13% (Nujiang), but the difference was not statistically significant (*P* > 0.05) (Table 1). The prevalence in different tissues varied from 10.98%% (liver) to 15.7% (tongue), however, there was no significant difference (*P* > 0.05) (Table 2).

**Table 1:** **Prevalence of *Toxoplamsa gondii *** **infection in cats detected by PCR in different cities in Yunnan province, southwestern China**

**Region**	**Total no.**	**Positive no.**	**Prevalence (%)**
Jinping	43	11	25.58
Nujiang	23	9	39.13
Kunming	4	1	25
Eryuan	54	8	14.81
Banna	13	4	30.77
Yimen	38	11	28.95
Total	175	44	25.14

**Table 2:** **Prevalence of *Toxoplasma gondii *** **infection in cats detected by PCR in different tissues**

**Tissues**	**Total no.**	**Positive no.**	**Prevalence (%)**
Brain	175	26	14.85
Liver	164	18	10.98
Heart	175	25	14.28
Tongue	172	27	15.7

Several studies have reported the *T. gondii* prevalence in cats in various regions in China, but little is known of the prevalence of *T. gondii* in cats in Yunnan province. A study reported an overall 21.3% seroprevalence of *T. gondii* among cats in Lanzhou, northwestern China [18]. The overall prevalence of *T. gondii* exposure in cats in Yunnan province was 25.14%, which is lower than that detected in Guangzhou (79.4%) [11]. The differences in prevalence of *T. gondii* exposure in cats in different provinces could be related to differences in ecological and geographical factors such as temperature, rainfall, or landscape differences. The methods used to determine *T. gondii* prevalence were also considered as a sophisticated factor to cause the differences.

Among the 44 B1 gene positive DNA samples, 13 of them gave complete genotyping results, three were genotyped at the 10 loci. Due to low DNA concentration, 28 of the 44 positive samples could not be genotyped and was therefore not used. Genetic characterization of the 16 samples revealed five genotypes, 11 of 16 samples were identified as ToxoDB#9, two of 16 samples may belong to genotype #225, one was Type II, one was ToxoDB#3, and one was ToxoDB#20. The results of genotyping of these strains and 9 references were summarized in Table 3. ToxoDB#9 was identified from 4 different cities of Yunnan province (Table 3), which suggests that this type is prevalent in this region. This same genotype was previously identified in cats in Beijing Municipality, Guangdong, Anhui, Guizhou, Shandong, and Hubei province [7-9,11,19], and it was also found in pigs in Guangdong, Henan, Yunnan and Anhui province [10,20-22]. Therefore, ToxoDB#9 is a predominant lineage prevalent in Mainland China. Many studies indicated that ToxoDB#9 has been isolated in North and South America [5,23-25], as well as other Asian countries, such as Sri Lanka, Vietnam [26,27], indicating that it has a worldwide distribution.

In this study, ToxoDB#3 (the type II variant) was identified for the first time in cats in China. This type had been founded from sheep in Qinghai province [10], from birds in Xinjiang Uygur Autonomous Region [28], from sparrows in Lanzhou, Gansu province [29], and from pigs in Zhongshan, Guangdong province [30]. ToxoDB#1 (the type II) was reported in humans [19], but this is the first report of this genotype in cats. We also founded ToxoDB#20 from one cat in Jinping, which is the first time that this has been reported in China too. Genotype #20 has been reported in dogs from Sri Lanka [27], feral cats in Egypt [31], stray dogs in Egypt [32], feral cats in Ethiopia [33], and sand cats in Qatar [34], indicating its wide spread from Africa to Asia. In this study we also identified a genotype most likely to be the #225, which has been reported from chickens in China [8]. Unfortunately, the number of cat samples is small in this study, especially in Kunming, where only 4 cats were collected, due to difficulties in collection of stray cats. To obtain more accurate information about the genetic diversity of *T. gondii* in cats in this unique province, more samples from more regions in Yunnan province should be used in further studies including the serology and bioassays. The conventional multilocus PCR-RFLP method relies on single-copy polymorphic DNA sequences, and usually a relatively higher amount of starting templates from the parasite is required. Due to the low DNA concentration of some samples, some *T. gondii* positive samples could not be completely genotyped.

Based on these results, the genetic diversity of *T. gondii* is quite high in cats in Yunnan province. Yunnan has a generally mild climate with pleasant and fair weather because of the province’s location on south-facing mountain slopes, receiving the influence of both the Pacific and Indian Oceans, it is China’s most diverse province, biologically as well as culturally. The province contains snow-capped mountains and true tropical environments, thus supporting an unusually full spectrum of species and vegetation types. This diversity could be related to the diverse climate and biology.

**Table 3:** **Summary of genotyping of *Toxoplasma gondii *** **in stray cats in Yunnan Province (Yn), southwestern China**

**Isolate ID**	**Host**	**Tissue**	**Location**	**SAG1**	**5′ + 3′ SAG2**	**Alt. SAG2**	**SAG3**	**BTUB**	**GRA6**	**c22-8**	**c29-2**	**L358**	**PK1**	**Apico**	**Genotype**
GT1	Goat		United States	I	I	I	I	I	I	I	I	I	I	I	Reference, Type I, ToxoDB #10
PTG	Sheep		United States	II/III	II	II	II	II	II	II	II	II	II	II	Reference, Type II, ToxoDB #1
CTG	Cat		United States	II/III	III	III	III	III	III	III	III	III	III	III	Reference, Type III, ToxoDB #2
MAS	Human		France	u-1	I	II	III	III	III	u-1	I	I	III	I	Reference, ToxoDB #17
TgCgCa1	Cougar		Canada	I	II	II	III	II	II	II	u-1	I	u-2	I	Reference, ToxoDB #66
TgCatBr5	Cat		Brazil	I	III	III	III	III	III	I	I	I	u-1	I	Reference, ToxoDB #19
TgWtdSc40	W-t deer		USA	u-1	II	II	II	II	II	II	II	I	II	I	Reference, ToxoDB #5
TgCatBr64	Cat		Brazil	I	I	u-1	III	III	III	u-1	I	III	III	I	Reference, ToxoDB #111
TgToucan	Toucan		Costa Rica	u-1	I	II	III	I	III	u-2	I	I	III	I	Reference, ToxoDB #52
TgCYn1	Cat	Heart	Eryuan, Yn	u-1	II	II	III	III	II	II	III	II	II	I	ToxoDB #9
TgCYn2	Cat	Heart	Eryuan, Yn	u-1	II	II	III	III	II	II	III	II	II	I	ToxoDB #9
TgCYn3	Cat	Heart	Kunming, Yn	u-1	II	II	III	III	II	II	III	II	II	I	ToxoDB #9
TgCYn4	Cat	Heart	Yimen, Yn	II/III	II	II	II	II	II	II	II	II	II	I	Type II, ToxoDB #3
TgCYn5	Cat	Liver	Jinping, Yn	u-1	II	II	III	III	II	II	III	II	II	I	ToxoDB #9
TgCYn6	Cat	Heart	Jinping, Yn	u-1	II	II	III	III	II	II	III	II	u-2	I	ToxoDB #20
TgCYn7	Cat	Tongue	Jinping, Yn	II/III	II	II	II	II	II	II	II	II	II	II	Type II, ToxoDB #1
TgCYn8	Cat	Tongue	Jinping, Yn	u-1	II	II	III	III	II	II	III	II	II	I	ToxoDB #9
TgCYn9	Cat	Heart	Nujiang, Yn	u-1	II	II	III	III	II	II	III	II	III	I	ToxoDB #9
TgCYn10	Cat	Tongue	Nujiang, Yn	nd	I	I	III	I	I	I	I	I	I	I	Type I variant 1
TgCYn11	Cat	Brain	Nujiang, Yn	u-1	II	II	III	III	II	II	III	II	III	I	ToxoDB #9
TgCYn12	Cat	Brain	Nujiang, Yn	u-1	II	II	III	III	II	II	III	II	III	I	ToxoDB #9
TgCYn13	Cat	Heart	Nujiang, Yn	u-1	II	II	IIII	III	II	II	III	II	III	I	ToxoDB #9
TgCYn14	Cat	Tongue	Nujiang, Yn	u-1	II	II	III	III	II	nd	III	II	III	I	ToxoDB #9
TgCYn16	Cat	Brain	Jinping, Yn	u-1	II	I	III	III	II	II	III	II	nd	I	ToxoDB #9
TgCYn17	Cat	Heart	Banna, Yn	I	I	I	III	I	nd	I	I	I	I	I	Type I variant 1

u-1 and u-2 represent unique RFLP genotypes, respectively; nd = no data.

## Conclusion

In conclusion, the results of the present study revealed a wide distribution of *T. gondii* infection in cats in Yunnan province, which may pose significant public health concerns. To our knowledge, this is the first report of *T. gondii* prevalence and genotypes in cats in southwestern China, and the first report of Type II *T. gondii* from cats in China.

## Competing interests

The authors declare that they have no competing interests.

## Authors’ contributions

FCZ and XQZ conceived and designed the study, and critically revised the manuscript. YMT, SYH, QM and HHJ performed the experiments, analyzed the data and drafted the manuscript. JFY and CS helped in study design, study implementation and manuscript revision. All authors read and approved the final manuscript.
